# Actin polymerization and depolymerization in developing vertebrates

**DOI:** 10.3389/fphys.2023.1213668

**Published:** 2023-09-08

**Authors:** Yang Bai, Feng Zhao, Tingting Wu, Fangchun Chen, Xiaoxiao Pang

**Affiliations:** ^1^ Stomatological Hospital of Chongqing Medical University, Chongqing, China; ^2^ Chongqing Key Laboratory of Oral Diseases and Biomedical Sciences, Chongqing, China; ^3^ Chongqing Municipal Key Laboratory of Oral Biomedical Engineering of Higher Education, Chongqing, China

**Keywords:** F-actin, G-actin, embryonic development, morphogenesis, organogenesis

## Abstract

Development is a complex process that occurs throughout the life cycle. F-actin, a major component of the cytoskeleton, is essential for the morphogenesis of tissues and organs during development. F-actin is formed by the polymerization of G-actin, and the dynamic balance of polymerization and depolymerization ensures proper cellular function. Disruption of this balance results in various abnormalities and defects or even embryonic lethality. Here, we reviewed recent findings on the structure of G-actin and F-actin and the polymerization of G-actin to F-actin. We also focused on the functions of actin isoforms and the underlying mechanisms of actin polymerization/depolymerization in cellular and organic morphogenesis during development. This information will extend our understanding of the role of actin polymerization in the physiologic or pathologic processes during development and may open new avenues for developing therapeutics for embryonic developmental abnormalities or tissue regeneration.

## 1 Introduction

Mammalian embryonic development begins with a single-celled fertilized egg, which proliferates and differentiates into billions of cells over time, forming tissues and organs and eventually complete individuals (Mittnenzweig et al., 2021). Many complex cellular activities, including deformation, adhesion, and movement (Wang S. et al., 2021; Gjorevski et al., 2022), regulate the morphogenesis and status of tissues and organs throughout embryonic development, and these activities occur in a specific space and time to meet the needs of tissue and organ shaping (Gillard and Roper, 2020). However, abnormal cell function may lead to wrong “development,” such as developmental defects and tumorigenesis (Lim and Plachta, 2021a). For example, the FoxD3, Sox10, and Ets1 genes originating from early proto-intestinal embryos in the neural crest cells (NCCs) activate them to initiate the epithelial–mesenchymal transition (EMT), thereby prompting cell migration to specific locations in the early embryo and inducing cell differentiation that contributes to the morphogenesis of various tissues and organs (Cheung et al., 2005; Simoes-Costa and Bronner, 2016; Martik and Bronner, 2017). The defects in the formation of the neural crest and its derivatives result in many congenital disabilities (Vega-Lopez et al., 2018).

Cytoskeleton is a flexible and dynamic structural system found in eukaryotic cells; it is crucial to maintain cell morphology and exert physiological functions during development (Clarke and Martin, 2021). It is composed of microtubules, microfilaments, and intermediate filaments (Hasan et al., 2022). In the early stages of embryogenesis, the morphological changes of cells and embryos are often accompanied by abundant cytoskeleton remodeling (Gillard and Roper, 2020; Lim and Plachta, 2021b). Actin is the major component of the cytoskeleton and is mainly found in the cytoplasm; it is divided into monomers [globular (G)-actin] and multimers [filamentous (F)-actin] (Dominguez and Holmes, 2011). Monomeric actin has six highly homologous isoforms, including skeletal muscle α-actin (ACTA1), cardiac muscle α-actin (ACTC1), smooth muscle α-actin (ACTA2), cytosolic β-actin (ACTB), cytosolic γ-actin (ACTG1), and smooth muscle γ-actin (ACTG2) (Fan et al., 2019). These homologous isoforms, encoded by different genes, are expressed at different concentrations in tissues and organs throughout the body (Herman, 1993). G-actin undergoes activation, nucleation, and elongation to polymerize into F-actin, which has a structural, mechanical, and modulatory role. F-actin participates in tissue and organ morphogenesis by regulating various cellular activities, such as cell adhesion, cell deformation, cell migration, and EMT (Schaks et al., 2019; Le et al., 2020).

Here, we reviewed the structural and molecular patterns of actin assembly and disassembly and described the role of actin polymerization and depolymerization in the major physiological activities occurring in various cell types. We also summarized the developmental defects associated with abnormal G-actin isoforms and defective actin polymerization. Finally, we concluded that actin polymerization and depolymerization are crucial for cellular contractility and are essential for the accurate embryonic development.

## 2 Structural and molecular mechanisms of actin polymerization

### 2.1 Structure of G-actin

**FIGURE 1:**
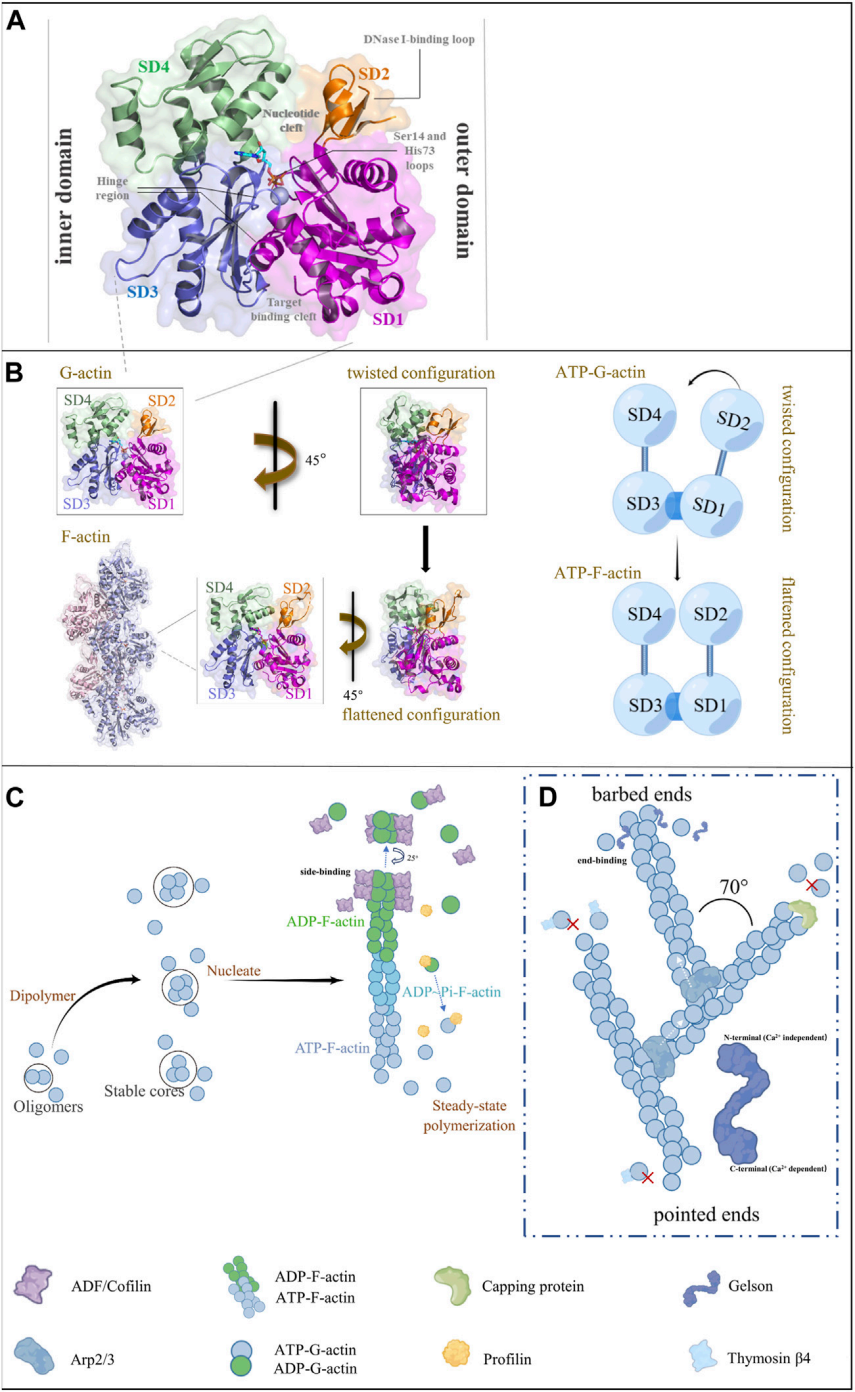
Actin structure and polymerization pattern. **(A)** Crystal structure of the G-actin. The structure of native G-actin was retrieved from Protein Data Bank (PDB; ID: 3 hbt). The G-actin monomer contains four subdomains (SD1, SD2, SD3, and SD4) and the loop is centered at residue Lys336. The linker helix at residues Gln137–Ser145 functions as an axis of a hinge connecting the two major domains of actin, consequently forming two clefts between the domains. The upper cleft binds the nucleotide, whereas the lower cleft is the binding site for ABPs. **(B)** The structure of F-actin was retrieved from PDB (ID: 8a2r). F-actin is a double-stranded and right-handed helix. Actin flattening in subdomain 1 and 2 during the G-to F-actin transition is shown in crystal or cartoon forms. **(C)** Actin polymerization and depolymerization, in which ATP-G-actin is involved in polymerization into ATP-F-actin, and ATP-F-actin in actin filaments spontaneously dissociates into ADP ∼ Pi-F-actin. It then becomes ADP-F-actin and finally depolymerizes into ADP-G-actin to achieve the balance of polymerization and depolymerization. **(D)** Barbed ends of actin polymerize faster and the tips polymerize slower. The new branches of actin filaments form a 70° angle with the old filaments (Figures C and D demonstrate the functions of the six ABPs).

Actin was discovered and named in the 1940s by a Hungarian biochemist Bruno Straub for its ability to activate ATP hydrolysis catalyzed by myosin (Straub, 1942; Glyakina and Galzitskaya, 2020). Actin in vertebrates exists in α, β, and γ isoforms (Dominguez and Holmes, 2011), and its monomer (G-actin) contains a 42 kDa core protein of 375 amino acid residues with various post-translational modifications, such as reacetylated N-terminal aspartic acid in the skeletal muscle α-actin (Herman, 1993; Dominguez and Holmes, 2011). The amino acid sequence in different actin isoforms is highly conserved with a few substitutions. Most of the substitutions occur at the N-terminus, which does not belong to the actin core (Dominguez and Holmes, 2011; Glyakina and Galzitskaya, 2020). Since the identification of the first G-actin crystal structure with DNase I in 1990 (Glyakina et al., 2020), over 100 structures of actin have been obtained (Glyakina and Galzitskaya, 2020). In a recent structural alignment study, the authors reviewed 72 G-actin structures reported from 1991 to 2020 and found that the crystal actin monomer structures were very similar (Glyakina and Galzitskaya, 2020). G-actin is divided into outer and inner domains, also known as small and large domains denoting their sizes in electron microscopic images (Dominguez and Holmes, 2011; Glyakina et al., 2020). Each domain contains two subdomains; the outer (small) domain consists of subdomains 1 (residues 1–32, 70–144, and 338–372) and 2 (residues 33–69), and the inner (large) domain comprises subdomains 3 (residues 145–180 and 270–337) and 4 (residues 181–269) (Dominguez and Holmes, 2011; Glyakina et al., 2020; Glyakina and Galzitskaya, 2020). The loop centered at residue Lys336 and the linker helix at residues Gln137–Ser145 function as an axis of a hinge connecting the two major domains, thereby forming two clefts between the domains. The upper cleft binds the nucleotide and associated divalent cations (Mg^2+^/Ca^2+^), whereas the lower cleft (mainly comprised of hydrophobic residues) contains the major binding sites for actin-binding proteins (ABPs) and participates in the regulation of longitudinal contacts between actin subunits in the filament (Kabsch et al., 1990; Fujii et al., 2010) (Figure 1A). G-actin is not an effective ATPase and exists in an ADP-bound state, whereas most crystal structures of F-actin have been solved in an ATP-bound state. The primary differences between ATP- and ADP-bound states of G-actin occur in the Ser14 β-hairpin loop and the sensor loop carrying methylated His73 (Oda et al., 2009; Dominguez and Holmes, 2011). The structure of native G-actin shows two major discrepancies compared to that of actin monomers in the filament (Graceffa and Dominguez, 2003). First, the D-loop located at the top of subdomain 2 (which mediates interactions with DNase I) is disordered in the native condition, whereas it has a structured conformation in the filamentous form. Second, subdomains 1 and 2 show a twisted arrangement in the native protein, whereas they have a flattened conformation in the filamentous form.

### 2.2 Structure of F-actin

Most G-actin structures have been determined using X-ray crystallography, whereas the structural models of F-actin have been obtained using other methods because filamentous form resists crystallization for a prolonged duration (Dominguez and Holmes, 2011). The first near-atomic resolution structure of F-actin—obtained by combining fiber diffraction with the G-actin structure—revealed that F-actin is a double-stranded and right-handed helix (Wang et al., 2010). The first complete atomic model of F-actin in complex with tropomyosin (an ABP) was obtained using cryo-electron microscopy (cryo-EM) in 2015 (Holmes et al., 1990). Several F-actin structures have been obtained using cryo-EM in all nucleotide states (Von der Ecken et al., 2015; Merino et al., 2018) and as complexes with diverse ABPs (Jvd et al., 2016; Mentes et al., 2018; Tanaka et al., 2018; Chou and Pollard, 2019; Huehn et al., 2020); however, details are inadequate because of moderate resolutions (approximately 3–4.5 Å) in all published F-actin structures (Pospich et al., 2021). Recently, Oosterheert et al. reported a detailed cryo-EM structure of F-actin in all nucleotide states polymerized in the presence of Mg^2+^ or Ca^2+^ at the resolution of approximately 2.2 Å (Oosterheert et al., 2022), this high-resolution structure provides more detailed information, including the visualization of hundreds of water molecules surrounding the F-actin filament (Oosterheert et al., 2022). The differences in the water position in the nucleotide-binding pocket provided a likely explanation for Mg^2+^-actin showing a faster polymerization rate than that of Ca^2+^-actin. The F-actin conformation for releasing inorganic phosphate was in a transient state. Q137 and its surrounding residues were the key factors sensing the nucleotide state after ATP hydrolysis and inorganic phosphate release and transmitting it to the periphery. The researchers suggested that F-actin in different states showed similar bulk structures, including the ADP-state, which challenges the previous concept of a destabilized ADP-state characterized by large amino acid rearrangements (Oztug Durer et al., 2010; Pivovarova et al., 2010). However, the structure of the F-actin state for releasing inorganic phosphate is still not available, and it is a challenge to capture this transient and high-energy state. Structural domains 1 and 2 produce a counterclockwise (45°) rotation around the hinge and become flattened during the conversion of G-actin to F-actin, (Figure 1B). In addition, some researchers have suggested that the current double-stranded F-actin structure maybe not the only one (Durer et al., 2010; Glyakina and Galzitskaya, 2020). Therefore, the complex organization of actin fibrils has not been fully explained, and new approaches and techniques are required to extend our knowledge in this context.

### 2.3 Polymerization and depolymerization of actin

The major function of actin relies on the actin filaments (F-actin) assembled from actin monomers, and large reserves of monomers (G-actin) are important to maintain the dynamic properties of actin filaments in cells (Skruber et al., 2018; Svitkina, 2018). Therefore, the dynamic balance between polymerization and depolymerization of actin is essential for various physiological functions in the cell, including proliferation, motility, shape, and polarity (Blanchoin et al., 2014; Gibieža and Petrikaitė, 2021; Xie et al., 2021). F-actin and myosin interact through cross-bridges to enable muscle contraction (Rayment et al., 1993). G-actin polymerization into F-actin is divided into three stages, including activation, nucleation, and elongation (Steinmetz et al., 1997). First, G-actin aggregates into small and unstable oligomers, and then the oligomers reach a length of approximately 3–4 subunits to form a stable core. Second, G-actin accumulates at both ends of the core and elongates into a filamentous polymer. Finally, ATP-G-actin polymerizes to form ATP-F-actin; the process requires ATPase-mediated ATP hydrolysis (Small et al., 2002). The catalytic rate of ATPase in free G-actin is low (Rould et al., 2006). After polymerization, the ATPase activity of actin increases, triggering nucleotide hydrolysis and subsequent release of phosphate (Blanchoin and Pollard, 2002). Therefore, ATP-F-actin dissociates into ADP ∼ Pi-F-actin by ATP hydrolysis. ADP-bound F-actin is less stable than ADP-bound G-actin, allowing easy disassembly of actin filaments. The ratio of G-actin to F-actin is gradually balanced and actin filament polymerization finally reaches a steady state, which contributes to the dynamic cyclic process of actin depolymerization in the cell (Bindschadler et al., 2004) (Figure 1C). Actin polymerization occurs mainly at the free membrane at the cell front (free F-actin network), where polymerization is faster and has a clear direction of movement; however, it could also occur at the cell-wall interface (adjacent F-actin network), where polymerization is slower. The adjacent F-actin network compresses the free F-actin network during mesenchymal cell migration, preventing its reverse flow and converting new aggregates into plasma membrane protrusions (Wilson et al., 2013).

The equilibrium of actin polymerization/depolymerization in cells is not stable and can change in response to external stimuli for adapting to environmental variations. For example, cellular lamellar lipid membranes composed of a network of actin filaments are highly dynamic structures in which individual filaments have a 10 s lifespan to ensure that cells can change their shape within minutes by polymerizing and depolymerizing actin (Salbreux et al., 2012). The rapid polymerization of actin helps to form fast and extended actin-based protrusion in the embryonic epithelial cells, promoting the rapid clearance of apoptotic targets through actin-related protein (Arp)2/3-dependent mechanical pushing. This process is important for early embryogenesis because sporadic cell death is a likely cause of developmental failure in early embryogenesis. This finding indicates that actin polymerization may regulate epithelial tissue clearance to facilitate error correction, which is relevant to the developmental robustness and survival of the embryo (Hoijman et al., 2021).

F-actin has barbed and pointed ends with different polarities during polymerization. Both ends can polymerize and depolymerize actin monomers; however, the barbed ends show faster rates of polymerization and depolymerization than that of the pointed ends (Kun-Kun and Yi, 2016). ATP-G-actin tends to polymerize at the barbed end, whereas ADP-G-actin tends to dissociate at the pointed end (Dominguez and Holmes, 2011) (Figure 1D). This process is called “treadmilling” (Phillips et al., 2012). ATP hydrolysis on actin is critical for regulating treadmilling. It is possible to regulate barbed end dynamics and filament length at a steady state, as well as to specify the functional interactions of actin with essential regulatory proteins, such as profilin and actin-depolymerization factor (ADF)/cofilin. However, the detailed molecular mechanism regarding ATP hydrolysis in F-actin depolymerization remains undetermined (Narita et al., 2011). Once the rates of depolymerization and polymerization are equal, the length of the actin filament is determined, and the process moves in the direction of polymerization (Merino et al., 2020). Some scholars used cryo-EM and found that the structure of the pointed end of the filament is different from that of the core part (Narita et al., 2011). However, the structure of the barbed end has not been reported (Merino et al., 2020). Notably, the total mass of the entire actin filament did not change during this period. Overall, the stable equilibrium between G-actin and F-actin is tightly regulated by various mechanisms to achieve different actin distributions and functions (Pollard, 2016).

Proteins, such as Arp2/3 complexes and formins, containing the WH2 structural domain (the most abundant actin-binding motif) promote the nucleation of ATP-bound actin monomers during the early stages of polymerization (Valencia and Quinlan, 2021). Subsequent extension of nascent filaments is achieved by formin, Ena/VASP proteins family, or proteins containing the polypeptide-WH2 (P-WH2) module. Filament growth can be terminated by capping proteins. Then, the most critical step in the actin polymerization is the formation of trimers or tetramers, during which the Arp2/3 complex drives the effective formation of daughter actin filaments from actin monomers, generating a large number of branched actin networks (Rotty et al., 2013). Formin family proteins bind to barbed ends and use their conserved formin homology 2 (FH2) structural domain to stabilize actin dimers and further recruit more monomeric actin (Breitsprecher and Goode, 2013). Moreover, Ena/VASP acts as an anti-capping protein to reduce the branching density of filaments by competing with Arp2/3 for binding to actin monomers (Applewhite et al., 2007; Bergeron et al., 2010).

The polymerization rate seems to depend on the type of G-actin. Different G-actin types show different polymerization rates, leading to different stability, elongation, and turnover rate of F-actin (Khaitlina, 1986; Khaitlina and Hinssen, 2008; Bergeron et al., 2010). However, this phenomenon has not been explored further, and its effects on embryonic development are unclear. Non-muscle actin shows some relative expression but does not participate in the tissue formation in myofibrils. Moreover, muscle actin and non-muscle actin show different intracellular localization and marked amino acid differences in muscle cells (Kaech et al., 1997a; Mounier et al., 1997). These findings suggest that these two types of actin cannot polymerize into the same type of F-actin (Müller et al., 2013; Kashina, 2020). Some authors have compared the polymerization of different isoforms of monomeric actin (Khaitlina, 1986; Bergeron et al., 2010). ACTC1 copolymerized with ACTA2 *in vitro*, whereas ACTB and ACTG1 did not. The differential localization of ACTA1, ACTA2, ACTC1, and ACTG2 isoforms suggested that they may not copolymerize in cells. For example, ACTC1 was localized to the sarcomere of cardiac (and muscle) cells, and coexpression of ACTA1 with ACTC1 or ACTA2 with ACTG2 did not affect the localization of sarcomeric proteins (Von Arx et al., 1995; Kaech et al., 1997b). Allen et al. conducted rheology experiments for testing the polymer stability of different G-actin formations and found that ACTA1 gels were the most elastic, and smooth muscle ACTB and ACTG2 gels were the least elastic. Cytoplasmic ACTB did not form any elastic gels (Allen et al., 1996). In addition, the polymers formed by β-actin and γ-actin were less stable than those formed by α-actin of the sarcomere, and in yeast, this lower stability of F-actin caused filament breakage (Khaitlina, 2001).

## 3 Actin-binding proteins

Eukaryotic cells have a functionally rich actin cytoskeleton regulated by several ABPs compared with prokaryotic cells (Blanchoin et al., 2014). Several hundred ABPs have been reported to date, including ADF/cofilin, profilin, gelsolin, thymosin β4, capping proteins, and the Arp2/3 complex. These ABPs bind in the target-binding cleft of actin monomers through side-binding, cross-linking, and end-binding. Side-binding and end-binding are the main binding modes involved in polymerization and depolymerization (Tokuraku et al., 2020) The G-actin polymerization rate is < 1 monomer/s *in vitro* and can exceed 1,000 monomers *in vivo* (Funk et al., 2019); therefore, the assembly from G-actin to F-actin is tightly controlled by multiple ABPs (Merino et al., 2020). Notably, most unpolymerized G-actin is bound to profilin and thymosin β4, preventing their irregular and spontaneous assembly *in vivo* (Kaiser et al., 1999). These ABPs are controlled by a small GTPase (RhoA/Rac1/Cdc42) signaling system that ensures actin assembly and disassembly in the cytoplasm at the correct time and space and determines the ultimate function of actin (Kadzik et al., 2020; Vakhrusheva et al., 2022). Here, we reviewed the role of some ABPs, including ADF/cofilin, profilin, gelsolin, thymosin β4, capping proteins, and Arp2/3 complex, in actin polymerization and depolymerization.

### 3.1 ADF/cofilin family

ADF/cofilin was first identified and purified from chicken embryo brain extract by Bamburg in 1980 (Bamburg et al., 1999). The ADF/cofilin family includes ADF, cofilin-1 (non-muscle cells), and cofilin-2 (muscle cells) (Kanellos and Frame, 2016). ADF/cofilin is regulated by LIM structural domain kinase and slingshot phosphatase, which phosphorylate/inactivate and dephosphorylate/activate ADF/cofilin, respectively (Ben Zablah et al., 2020). Cofilin-1 isoforms are predominantly expressed in mammalian neuronal development (Bamburg et al., 2021), whereas the myotype cofilin-2 is mainly expressed in transverse muscle and plays a critical role in regulating the assembly of skeletal muscle contractile structures (Mohri et al., 2019). Structurally, Cofilin locates at the interface of two actin subunits within a chain, which binds preferentially to ADP-bound F-actin and controls actin depolymerization in a pH-sensitive manner(Figure 1C). At lower pH, actin filaments have higher rates of polymerization and depolymerization. When the filaments are saturated with ADF/cof1, the rate of depolymerization of saturated filaments decreases compared to bare filaments, but the rate gradually increases with increasing pH. (Wioland et al., 2019). Cofilin binds to the barbed ends of actin filaments by side-binding, thereby exposing and turning the helix within the F-actin cluster and eventually severing the filament (Figure 1C) (Galkin et al., 2011; Merino et al., 2020). High-speed atomic force microscopic images revealed that the actin filaments bound to the cofilin cluster were hypertwisted by 25%, while the exposed region of the cofilin cluster faced the pointed end of the F-actin. The filament was hypertwisted at approximately half-helix spacing (containing 14 actin monomers) (Galkin et al., 2011). In animal cells, cofilin is localized to the platelet at the front of the cell, and sufficient G-actin is present to ensure continuous polymerization at the front of keratinocytes, neural crest-derived cells, and macrophages to generate thrust, which is important for cell migration (Aizawa et al., 1997). ADF/cofilin maintained axonal regeneration after spinal cord injury by increasing the turnover rate of actin during neuronal development and regeneration (Tedeschi et al., 2019). In addition, cofilin-2-deficient mice displayed disordered and scattered filament structures in the cytoplasm of cardiomyocytes, resulting in structural defects in cardiac fibers (Mohri et al., 2019). Taken together, cofilin has a crucial role in regulating the dynamics of actin filaments; therefore, it may have an important role in the pathophysiology of cancer, neurodegenerative diseases, and congenital muscle development defects.

### 3.2 Profilin family

Profilin was first discovered over 40 years ago, and four isoforms have been identified to date, including Pfn1, Pfn2, Pfn3, and Pfn4. The protein (molecular weight: approximately 19 kDa) can inhibit actin polymerization *in vitro* (Carlsson et al., 1977) and is largely expressed in the brain, heart, kidney, liver, muscle, and testis (Tariq et al., 2016; Karlsson and Dráber, 2021). Profilin can convert ADP-G-actin released from filaments into ATP-G-actin (Goldschmidt-Clermont et al., 1991) (Figure 1C). It can inhibit the spontaneous nucleation of actin and thus repress the elongation of F-actin (Yarmola and Bubb, 2006). Notably, the coexistence of profilin and ADF/cofilin increased actin turnover; profilin prompted the conversion of ADP-G-actin to ATP-G-actin and added it to the barbed ends, cofilin simultaneously dissociated ADP-F-actin from the pointed ends (Didry et al., 1998). This process enhanced the intracellular recycling rate of G-actin. Pfn1-deficient mice showed reduced brain volume and cerebellar hypoplasia after birth, which appeared to be caused by defects in basal glial cell division in the brain resulting from abnormal accumulation of actin filaments in development (Chédotal, 2010). In contrast, defects in Pfn2 did not cause structural changes in the brain but caused neuronal axon growth defects. Notably, this phenotype was partially rescued by Pfn1 overexpression (Michaelsen et al., 2010). However, to date, there are few authors have elaborated studies on the role of profilin in organ development, and its functional mechanism during development needs to be further studied.

### 3.3 Gelsolin superfamily

Gelsolin is one of the most abundant ABPs with a molecular weight of 80 kDa; it plays a critical role in cell movement, shape, and metabolism (Feldt et al., 2019). High levels of gelsolin expression were found in the lungs and heart, whereas low levels of GSN expression occurred in skeletal muscle, testicles, and kidneys (Arai and Kwiatkowski, 1999). Gelsolin is composed of six domains, which can severe actin filaments (Nag et al., 2013). It can cut the actin filaments released by dead cells in the plasma, thereby boosting metabolism (Takeda et al., 2020). Gelsolin severs actin filaments in a Ca^2+^-dependent manner and coats the barbed ends of F-actin (Takeda et al., 2020). The amino-terminal of gelsolin binds to two actin monomers, and F-actin can be severed without free calcium. In contrast, the carboxyl-terminal only binds to a single actin, depending on free calcium (McLaughlin et al., 1993) (Figure 1D). Interestingly, ADF/cofilin shares the actin-binding sites with gelsolin, which can both alter the F-actin conformation and severe it (Van Troys et al., 1997). However, ADF/cofilin has been considered the main regulatory protein of actin cytoskeletal recombination (Bamburg, 1999). Gsn^*−/−*^ mice were viable and fertile; however, platelet function, neutrophil chemotaxis, and fibroblast migration were reduced in the Gsn knock-out mice. Notably, excessive actin stress fibers were observed in the Gsn^*−/−*^ fibroblasts, and neither gelsolin nor other proteins with similar actin filament-severing activity were expressed in early embryonic cells. Therefore, gelsolin or other similar proteins regulating actin filament dynamics maybe not involved in the early embryogenesis (Huff et al., 2001; Silacci et al., 2004; Huang et al., 2016).

### 3.4 Thymosin β4

Thymosinβ4 (Tβ4) is a 43-amino acid protein abundant in the nervous tissue and cells of the circulatory system, such as platelets, white blood cells, and macrophages (except for red blood cells) (Huff et al., 2001). The Lys-18 site of Tβ4 intersects with one of the four amino-terminal acid residues of actin, and the carboxyl-terminal region of Tβ4 (Lys-38) cross-links with Gln-41 at the tip of G-actin subdomain 2 (dos Remedios et al., 2003). Tβ4 can prevent the spontaneous polymerization of actin by specifically binding ATP-G-actin at both ends of the filament (Xue et al., 2014) (Figure 1D). Therefore, it is crucial for actin-mediated cell migration and extracellular matrix (ECM) remodeling (Qiu et al., 2007; Xiang et al., 2023). Tβ4 mRNA is mainly expressed in hippocampal neurons, neocortices, amygdala, and oligodendrocytes (Carpintero et al., 1999); therefore, it may play a role in the production and remodeling of neurons. Tβ4 depletion obstructed cell migration and differentiation, leading to developmental defects in coronary arteries of developing mouse hearts (Smart et al., 2007). Tβ4 depletion induced the loss of actin- and adhesion junctions (AJ)-dependent adhesion in epidermal cells during mouse skin development, resulting in epidermal dysplasia accompanied by eyelid insufficiency and hair follicle dysplasia (Padmanabhan et al., 2020). Notably, Tβ4 and profilin can compete with each other to bind actin monomers because they share a common actin binding sequence (LKHAET) (Vancompernolle et al., 1991), which indicates that Tβ4 and profilin may coordinate to regulate the rate of actin assembly through competitive binding (Sun et al., 1996). However, the specific role of Tβ4 and profilin in the development remains elusive, and how they regulate the rate of actin assembly in response to changes in the external environment needs to be further investigated.

### 3.5 Capping proteins

Heterodimeric capping protein (CP/CapZ) is a conserved ABP. where CapZ is the most abundantly expressed capping protein with α1, α2, α3 and β subunits, encoded by Capza1, Capza2, Capza3 and Capzb, respectively (Edwards et al., 2014). The transverse muscle Z-disks is a multiprotein complex at the boundary between muscle segments and plays an essential role in maintaining the structure and function of the transverse muscle (Sheikh et al., 2007). Z-diskcs anchor actin-rich filaments and are responsible for maintaining mechanical stability within cardiac myocytes. CapZ is located at the spiny end of F-actin in the Z-disk to inhibit polymerization (Figure 1D), where it interacts with profilin, Arp2/3 complexes, and membrane-local nucleation promoter of Wiskott–Aldrich syndrome protein (WASP) to promote the formation of short filaments and branching networks in non-muscle cells (Akin and Mullins, 2008). This change assists the cytoplasmic actin polymerization in producing the force to push against the cell membrane (Bieling et al., 2016; Mueller et al., 2017). CapZ has been observed during the formation of filamentous and lamellar pseudopods during cell migration (Pocaterra et al., 2019). Knockout of CapZ increased the amount of F-actin in Hela cells and induced the accumulation of early endosomes (Wang D. et al., 2021), leading to autoimmune diseases, neurodegeneration, diabetes, and cancer (Parachoniak and Park, 2012; Mendoza et al., 2014). The inactivation of CapZ overactivated yes-associated protein (YAP), leading to organ overgrowth (Pocaterra et al., 2019). Capza2 gene mutation disrupted actin polymerization and nucleation during development, leading to nonsyndromic neurodevelopmental disorders in children (Huang et al., 2020). Therefore, CapZ is important for regulating the density of actin filaments in myogenic fibers.

### 3.6 Arp2/3 complex

The Arp2/3 complex is an ABP composed of Arp2, Arp3, and five smaller (Arc) proteins (Cossart, 2000). The main function of Arp2/3 is to promote the production of new branches of actin filaments (Mullins et al., 1998) (Figure 1D), which relies on its activation by nucleation-promoting factors, such as WASP, Scar/WAVE, WASH, and WHAMM (Buracco et al., 2019). For example, N-WASP-mediated activation of the Arp2/3 complex in Purkinje cells on the neuronal plasma membrane was essential for normal axon development (Pinyol et al., 2007). During early embryonic development, actin in epithelial cells rapidly expanded through Arp2/3 to form a long arm-like structure for rapid uptake and removal of apoptotic cells in tissues, which was conducive to the stability and survival of early embryos in the temporary absence of immune cells (Hoijman et al., 2021).

Overall, ABPs are the most important and direct class of regulators of actin polymerization and depolymerization. Disruption of actin polymerization caused by abnormal ABPs may induce cancer and autoimmune diseases. Individuals deficient in different ABPs show varying degrees of developmental defects and organ dysfunction (Saotome et al., 2004; Vauti et al., 2007; Wang et al., 2020). However, some researchers have also found an embryonic lethal phenotype after knocking out ABP-related genes, such as Arp3-deficient mice failing to develop to the blastocyst stage (Tocchetti et al., 2010) and ezrin-mutant mouse pups dying shortly after birth (Marston and Goldstein, 2006). Surprisingly, Eps8-deficient mice showed an improved overall metabolic state and a longer lifespan (Steinbacher and Ebnet, 2018), suggesting that ABPs have variable roles in the growth and development of the organism; however, the exact mechanisms need further exploration.

## 4 Actin is essential for cell physiological activities

### 4.1 Actin is a major component of adhesion junctions

Cellular behaviors that drive morphogenesis depend on the nature of biological forces during embryonic morphogenesis (Merle and Farge, 2018). Intracellular mechanical forces are transmitted to the local environment and extended to the entire tissue through intercellular and cell-matrix adhesions (Wozniak and Chen, 2009). Therefore, the ability of cells to sense, generate, and transmit mechanical signals is fundamental to guiding tissue and organ morphogenesis (Takeichi, 2014; Mege and Ishiyama, 2017). Adhesion junctions (AJs), one of the main cell–cell junctions in eukaryotes, are key components that promote epithelial and non-epithelial tissue stabilization and dynamic cell motility (Lecuit and Yap, 2015) by mediating cell adhesion, migration, and EMT.

**FIGURE 2:**
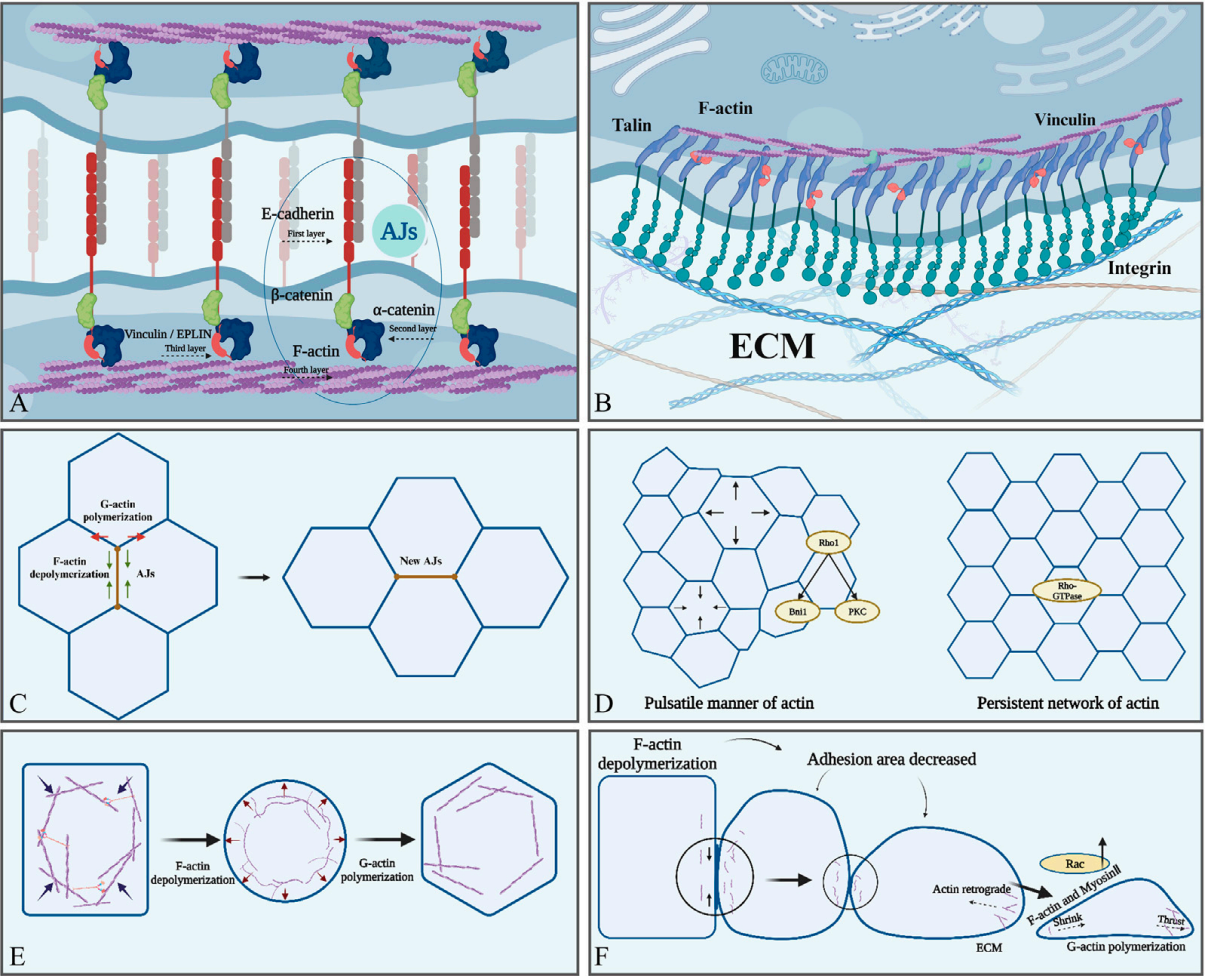
Actin is involved in various cellular physiological activities. **(A)** AJs form the intercellular junctions, and E-cadherin (red) form the intercellular contact. The cytoplasmic part of E-cadherin binds to β-catenin (green) and P120-catenin (red), and then β-catenin binds to α-catenin (blue). Finally, the -COOH terminus of α-catenin binds directly to F-actin (purple). **(B)** F-actin is indirectly attached to integrin via talin to ensure focal adhesion between cells and the ECM. **(C)** F-actin regulates the length of cell adhesion junctions and the formation of new adhesion junctions through polymerization and depolymerization. **(D)** Two types of actin aggregation: a pulsatile network and a persistent network. **(E)** The tendency of F-actin to depolymerize leads to increased cellular deformability, whereas the tendency to polymerize helps maintain cellular morphology. **(F)** The tendency of F-actin to depolymerize contributes to the reduction of adhesion between epithelial cells. The tendency to promote polymerization enhances the ability of cells to migrate and acquire a mesenchymal phenotype, in which actin polymerization at the anterior edge of migrating cells generates thrust, whereas actin filaments at the posterior edge generate contractile force together with myosin. Created with BioRender.com.

AJs are cell–cell adhesion structures dominated by the transmembrane protein E-cadherin. E-cadherin consists of an extracellular region with five cadherin repeats, a transmembrane structural domain, and a carboxyl-terminal cytoplasmic structural domain (Rimm et al., 1995). The integration of adhesion and contractility at E-cadherin junctions is a critical determinant of cell and tissue morphogenesis (Watabe-Uchida et al., 1998). The physical connection between the cadherin complex and contractile apparatus relies on the participation of F-actin, in which α-catenin plays a key role by directly binding to actin filaments (Abe and Takeichi, 2008) and assisting in interaction with α-catenin-associated proteins, such as vinculin (Hong et al., 2013) or EPLIN (Indra et al., 2020) (Figure 2A). Additionally, the lifespan of cadherin clusters is regulated by their α-catenin-mediated coupling to F-actin (Kanchanawong et al., 2010). Interestingly, E-cadherin can directly bind to F-actin in the punctate adherens junctions (Bertocchi et al., 2017).

Recently, some authors have proposed a four-layer structural model of the AJ. The first layer is the extracellular adhesion layer, where mechanosensitive elements regulating adhesion are present, and the second layer is near the cell membrane and further promotes adhesion. The third layer contains another mechanosensitive element whose main function is to regulate the cytoskeleton, and the last layer is the F-actin regulatory layer containing multiple ABPs that synergistically regulate the state of the cytoskeleton, such as binding, polymerization, and nucleation (Maître et al., 2012; Tang and Brieher, 2012; Mège and Ishiyama, 2017) (Figure 2A). Interestingly, when adhesion is impaired, it is usually not a break in extracellular adhesion but in the connection between the adhesion complex and actin cortical (Mascarenhas et al., 2022). This phenomenon was also supported by the observation that the adhesion complex showed a high degree of instability after F-actin uncoupling (Mitchison and Kirschner, 1988). Additionally, F-actin abnormalities disrupted endothelial cell adhesion (Elosegui-Artola et al., 2018), and aberrant F-actin reorganization and loss of intercellular adhesion were observed in uremic mediator-treated endothelial cells. This change was reversed by vitamin D treatment to restore vascular endothelial-cadherin expression. Moreover, abnormal or defective distribution of F-actin and G-actin disrupted the G/F-actin ratio, resulting in abnormal AJ stability or even loss of actin in keratin-forming cells (Padmanabhan et al., 2020). Overall, these findings indicated that F-actin is important for AJ assembly and stability.

Actin polymerization generates push or pull forces physically coupled to AJs, which propagate mechanical forces to neighboring cells or ECM (Arbore et al., 2022; Wang A. et al., 2022). The linker proteins, including α-catenin, β-catenin, and talin, play a critical role in mechanotransduction by integrating F-actin and cadherin complex (Case and Waterman, 2015; Elosegui-Artola et al., 2016). When an external force is applied to talin, a conformational change occurs and the binding site of the nexin is exposed, thereby enhancing its interaction with F-actin (del Rio et al., 2009; Owen et al., 2022; Huang et al., 2017). The C-terminal actin-binding site of talin, ABS3, binds to actin when it is subjected to external forces, and the interaction is dependent on the direction of the force, thereby promoting adhesive growth and cytoskeletal force production (Chandra et al., 2022), and vinculin acts as a force-sensing protein in this process (Li et al., 2020; Chandra et al., 2022). These results indicate that mechanosensation may be a universal property of the linker proteins involved in bridging the cytoskeleton to the cadherin complex.

Recently, a positive feedback loop between F-actin and cadherin-based adhesion has been identified. In this loop, E-cadherin-based adhesion signaling promotes the production of barbed ends and protrusions of F-actin at the leading edge of cells, which, in turn, facilitates the new AJ formation by reducing the space between neighboring cells (Santa-Cruz Mateos et al., 2020; Yu et al., 2022). The protrusions formed by actin polymerization are dynamic and vary in association with the accumulation of basal myosin and the periodicity of the contractile pulse of the cell membrane (Jodoin et al., 2015; Uechi et al., 2022). Aberrant actin protrusion formation reduces E-cadherin expression and the misalignment of myosin Ⅱ, ultimately affecting adhesion (Ikawa and Sugimura, 2018). Further, disturbing F-actin turnover by gene depletion or acute drug treatments breaks the interaction between the contractile actomyosin network and AJs (Romero et al., 2020; West and Harris, 2020), in which AIP1 and cofilin act as the promoting factors (Romero et al., 2020). This mechanism is important to understand how cells respond to and resist tissue stress during rearrangement. Promoting actin polymerization also enhanced tissue closure on the back of *Drosophila* during development (Kong et al., 2022). Moreover, actin polymerization serves as a safety mechanism for rapid repair when E-cadherin function is impaired, preventing further tearing of inter-tissue junctions by non-dependent muscle contractile forces (Yu et al., 2022).

The classical model of ECM adhesion is dependent on focal adhesion formed by the binding of integrins, actin, vinculin, and talin (Guilluy et al., 2011) (Figure 2B). ECM-regulated VE-calmodulin activates downstream integrins and focal adhesion kinase (FAK). Then, FAK couples integrins to the cytoskeleton and further recruits adhesion proteins and actin, promoting the formation of ECM adhesion. Simultaneously, these processes mechanically enhance calmodulin adhesion, creating a positive feedback loop (Bays et al., 2014); adhesion kinase, Abl kinase, and RhoA GTPase are the key components of this loop. FAK promotes the formation of cell–ECM focal adhesion, Abl recruits adhesion proteins, and RhoA regulates actin remodeling (Guilluy et al., 2011; Bays et al., 2014).

Cell–cell (Truong Quang et al., 2013; Wu et al., 2015; Indra et al., 2018) and cell–ECM (Changede et al., 2015) adhesions are composed of small 50–100 nm clusters of adhesion proteins. Actin can organize cadherins into large, micrometer-size clusters known as puncta; the process is dependent on the actin polymerization mediated by Arp2/3, Ena/VASP family members (EVL), and collapsin response mediator protein-1(CRMP-1) (Strale et al., 2015). Indrajyoti et al. further observed that the polymerization of actin regulated the assembly and disassembly of the cadherin cluster. The size of the cellular junctions can control the strength and stability of the adhesions; therefore, the role of actin polymerization in modulating the size of the cadherin cluster may contribute to adjusting the adhesion strength (Chandran et al., 2021). Notably, clustered E-cadherin has stronger mechanical connections to the actin cytoskeleton and resists higher tensions (Strale et al., 2015). Supported by actin, E-cadherin is regulated by changes in epithelial cell elasticity (Eftekharjoo et al., 2022).

### 4.2 Actin polymerization and depolymerization in cell deformation

Cell shape change or cell deformation is characterized by the variation in the length of the cell–cell junction and the surface area of the cells (Kong and Großhans, 2020). Thereby cooperating with the growth of complex tissue structures and shaping the organs (Heer and Martin, 2017). The classic example of cell deformation affecting embryonic development is the change in cell shape during *Drosophila* proto-intestine development, which drives the tissue to bend and fold inward to form mesodermal invaginations (Leptin and Grunewald, 1990; Sweeton et al., 1991). Additionally, cell shape changes are also critical for the development of *Drosophila* placental tissue (Sanchez-Corrales et al., 2018). Cardiac progenitor cells in the second heart region (SHF) of the mouse visceral mesoderm lay the foundation for subsequent cardiac morphogenesis by altering cell morphology, and morphological abnormalities in the SHF lead to defects in outflow tract development (Vincent and Buckingham, 2010). Therefore, understanding the mechanisms of cell deformation is fundamental to deciphering organ morphogenesis.

In a single cell, the nucleus senses external pressure and translates stress-induced shape changes into deformation signals that regulate cellular behavior to adapt to the microenvironment. This process is transient (<1 min) and is dependent on the cytoskeletal contraction regulated by actin polymerization and elevated expression of cortical myosin II (Venturini et al., 2020). In cell clusters, cell deformation is mainly dependent on AJs and cortical tension interactions (Serwane et al., 2017). The distribution of AJs across the plasma membrane during cell deformation is polarized and controlled by planar cell polarity, which coordinates the organizational remodeling of individual cells and cell populations (Halbleib et al., 2007; Gray et al., 2011). In contrast, cortical tension is produced by actin polymerization and myosin II synergistically (Yubero et al., 2020). The most prevalent shape change in cells is apical contraction driven by cortical actin, which reduces the cell area and causes the tissue to bend or involute (Svitkina, 2020). Actin depolymerization causes the cell to lose rigidity and shape, whereas polymerization allows the cell to acquire a new shape again. Actin has two modes of polymerization during this period. First, actin aggregates in a pulsatile manner, which is dependent on the formin-related gene (Rho1) (Chugh et al., 2017). Rho1 is a GTPase signaling protein that acts as a molecular switch and functions as a key regulator of the actin cytoskeleton by bridging integrator 1 and protein kinase C 1, which is critical for regulating actin polarization (Helliwell et al., 1998; Yayoshi-Yamamoto et al., 2000; Nomura and Inoue, 2019). Second, actin aggregates to form a persistent network regulated by the key nucleator Frl, which is activated by Rho GTPases (Rauzi et al., 2008) (Figure 2D). The persistent network is important for the transmission of forces between adjacent cells, and the ability to transmit contractile forces uniformly over long distances between cell clusters is dependent on this network (Dehapiot et al., 2020). Reduced expression of Frl diminishes epithelial cell deformability and leads to sparse intercellular adhesion junctions, thereby affecting the transmission of contractile forces to cell cluster boundaries, ultimately leading to defective tissue morphology (Irvine and Wieschaus, 1994).

In addition, the actin fiber network can provide mechanical force in real-time, which is a key factor affecting cellular deformation (Del Signore et al., 2018). The network is mainly composed of isotropic F-actin crosslinked with some ABPs (Svitkina, 2020). Actin produces protrusive force by forming branching and bundle structures, whereas contractile force is provided by forming bundles or loose tissue networks with non-muscle myosin II. This bidirectional force generated by actin can coexist in each cell (Ikawa and Sugimura, 2018) and mediate the strength of cell–cell adhesion junctions. Moreover, this force can control the stiffness, extension, and contraction of cell shape, finally providing the basis for cell migration and EMT (Wang et al., 1990). Actin exerts the thrust on the plasma membrane rather than the contractile force of actin-myosin (in muscle cells) during the deformation of non-muscle cells such as neutrophils (Herrera-Perez and Kasza, 2018). In contrast, actin filaments play an important role in generating contractile tension in epithelial cells (Fernandez-Gonzalez and Zallen, 2011). Further, the cells suffer from transient contraction to gradual stabilization, and then to irreversible deformation, which is often described as “oscillatory ratcheting” (Gorfinkiel and Blanchard, 2011; Gillard and Röper, 2020). Actin polymerization is the molecular basis of this “ratchet” mechanism, and the pulsatile activity of actin provides transient mechanical forces to promote changes in cell shape (Coravos et al., 2017). These changes were regulated by GTPases, RhoA, and Afadin (Lessey et al., 2012; Cavanaugh et al., 2020). The strong or prolonged RhoA activation could drive the connection beyond the deformation threshold, thereby driving irreversible connection shortening (Seltmann et al., 2013). Additionally, an increase in the deformability of keratin-forming cells with the inhibition of actin polymerization was observed, accompanied by a significant decrease in cell stiffness (Kunschmann et al., 2019). Kunschmann et al. (Barlan et al., 2017) corroborated the idea that disturbance of actin polymerization was responsible for the altered cell deformability, and Rac1 may be involved in the regulation of actin polymerization. These findings indicate that the protrusion force of the F-actin branch can balance the contraction force to produce proper cell shape change or stiffness and maintain cell–cell contact. This has important implications in the remodeling of epithelial sheet tissue during development, such as protointestinal embryo formation in mice and stretching of *Drosophila* oocytes (Behrndt et al., 2012; Cetera et al., 2014). Embryonic epithelial sheets with abnormal F-actin aggregation fail to stretch and spread efficiently, thereby impairing the further development of early protointestinal embryos (Maître and Heisenberg, 2013).

Taken together, AJ provides stable intercellular physical connections together with the actin network of neighboring cells, and the polymerized actin forms a branching network with myosin that adjusts cell stiffness and shape changes according to the rapidly changing microenvironment in morphogenesis (Irvine and Wieschaus, 1994; Keller, 2005; Leckband and de Rooij, 2014). Interestingly, most of the results of these studies mention the effects on migration in addition to those on the shape. These findings suggest that cell shape is usually required to produce effects on embryonic development in conjunction with migration; however, the separate effects of shape changes on embryonic development are unclear.

### 4.3 Actin polymerization and depolymerization in cell migration

Embryonic development requires directed cell migration in mammals. For example, epithelial cells migrate synergistically to form the proto-intestinal embryo, and precursor cells, including the NCCs and cells in the brain ventricles, leave their ecological niche and move toward their target site (Leptin, 2005; Richardson and Lehmann, 2010; Theveneau and Mayor, 2012; Myat et al., 2019). Therefore, cell migration is a fundamental biological process for embryogenesis and is a hallmark of individual or collective forms (Yang et al., 2019). Single-cell motility is the simplest form of cell migration, and the typical patterns are amoebic and mesenchymal motility (Shafqat-Abbasi et al., 2016). Moreover, single-cell migration in the two-dimensional plane is based on the protrusion of membrane lipids (Hervas-Raluy et al., 2019). Cell migration is dependent on the combined action of myosin and actin, and actin polymerization leads to leading-edge displacement and myosin forces mediate posterior contraction. Nucleus movement is dependent on cytoplasmic actin depolymerization (Tambe et al., 2011). In contrast, collective migration is the transfer of stress from neighboring cells through intercellular junctions. Cells move in the direction of minimal intercellular shear stress (Theveneau et al., 2010), which is important for the formation of complex tissue-organ structures by integrating the collective migration of epithelial and mesenchymal cells in embryonic tissues (Ananthakrishnan and Ehrlicher, 2007).

Epithelial and mesenchymal cells migrate through the “protrusion–adhesion–contraction” cycle (Jacquemet et al., 2017), during which pioneer cells use the laminae or filopodia to sense the ECM and direct the movement of the cell population (Angelini et al., 2011). Coherent migration in clusters or streams requires cells to move in the same direction simultaneously (Scarpa and Mayor, 2016); therefore, the anterior and posterior portions of the cluster must be mechanically connected to achieve successful migration of the posterior row of cells (Haeger et al., 2015). During this process, E-cadherin-mediated AJs are well-regulated in time and space (Mitchison and Kirschner, 1988), allowing cells to acquire sufficient plasticity for collective migration while maintaining sufficient intercellular adhesion to ensure tissue integrity. Actin polymerization plays an integral role in collective cell migration by regulating E-cadherin AJs, integrating forces generated by individual cells within tissues, and maintaining the integrity of cell–cell junctions (Angelini et al., 2011; Scarpa and Mayor, 2016; Jacquemet et al., 2017). Actin in the pioneer cells accumulates in the cortex at the leading edge of the cell, driving the protrusion of the plasma membrane and promoting the formation of focal adhesions of the cells toward the outer matrix (Jacquemet et al., 2017). Focal adhesions further associate with the lamellar actin network through the binding of actin to vinculin, talin, and α-actinin (Pollard and Borisy, 2003). A highly dense branching network of actin-containing structures extends at the cell periphery during the migration (Swaminathan et al., 2017). The barbed ends in this structure face toward the cell edge. Actin polymerization pushes the membrane forward, whereas the actin network pushes inward, producing a continuous centripetal motion known as actin retrograde flow (Figure 2F). Then, actin countercurrently attaches to the substrate via focal adhesion, causing the cell to move forward (Bodor et al., 2020). Simultaneously, posterior cell contraction and release of focal adhesions also contribute to the basic migration process (Maruthamuthu et al., 2010). Migration in a three-dimensional environment can be achieved without relying on outer matrix adhesion, and current studies have focused on suspended cells such as leukocytes (Mitchison and Kirschner, 1988; Bergert et al., 2015). However, non-specific matrix friction may be the source of the migratory force of cells as they pass through dense tissues during development (Bergert et al., 2015). Cells rely on nonspecific friction between extracellular matrices to generate traction forces in nonadhesive migration, which are much smaller than the focal adhesion of the outer matrix. Both F-actin and myosin are enriched in the posterior cortex of cells in this migration pattern; ablation of actin in the posterior reduces the rate of cell migration, whereas ablation at the anterior edge has no effect (Yamada et al., 2019). Therefore, F-actin and myosin may play a role in this pattern; however, the exact mechanism has not been studied in detail.

Actin polymerization is associated with various types of cell migration during tissue and organ development. For example, mesenchymal stem cell migration begins with cell polarization driven by local actin polymerization and is highly dependent on integrin- and actin-mediated cell contractility (Thomason et al., 2020). RhoA kinase mediates the formation of actin and myosin contraction fiber bundles (actomyosin filament bundles). Actomyosin contractility contributes to the establishment of a forward and backward polarity state during the migration process (Smith et al., 2020). Oligodendrocytes are essential for the development of central nervous system myelin sheaths, and their motor behaviors are mainly driven by actin assembly (Thomason et al., 2020). Primary cilia are microtubule-based organelles through which most mammalian cells receive and integrate mechanical and chemical signals from the extracellular environment (Waters and Beales, 2011; Smith et al., 2020). Excessive polymerization of actin may activate the YAP/TAZ pathway, leading to cilia breakage and causing kidney disease, blindness, obesity-related diseases, severe neurodevelopmental abnormalities, and skeletal dysplasia (Waters and Beales, 2011). F-actin depolymerization promotes the migration of cardiac microvascular endothelial cells during cardiac development, thereby facilitating cardiovascular generation (Wei et al., 2010).

Interestingly, abnormalities in the structure of actin can also affect cell migration. N-terminal-acetylation of actin subunits affected actin assembly *in vitro* (Arnesen et al., 2018). In addition, disruption of the SETD2–HTT–HIP1R axis inhibited actin methylation, leading to defective actin polymerization and impaired cell motility (Seervai et al., 2020). Therefore, actin polymerization is essential for cell migration during embryonic development. However, some questions are still unanswered. Cytoplasmic actin depolymerization is responsible for pulling the nucleus forward during migration but the role of nuclear actin in this process is unclear. Understanding the role of ABPs in actin migration will remain be an interesting research area in the future.

### 4.4 Actin polymerization and depolymerization in EMT

EMT is a process in which epithelial cells lose apical-basal polarity, lose intercellular adhesions, and acquire the morphology and characteristics of mesenchymal cells to facilitate migration using the ECM (Amack, 2021). EMT involves marked changes in cell morphology, adhesion, and migration (Milmoe and Tucker, 2021), and this transformation exists throughout the cell cycle and animal development from embryo to death (Li et al., 2021). EMT is involved in the formation of endoderm, mesoderm, NCCs, and heart valves in embryonic development (Li et al., 2021), and abnormal EMT may cause embryonic dysplasia during development (Li et al., 2021). For example, epicardial cells during heart development promote organ morphogenesis through EMT and migrate into the myocardium, and disruption of epicardial cell EMT leads to embryonic lethality in mice (Sun et al., 2021).

Cell–cell junctions are a classical characteristic of epithelial cells (Pinheiro and Bellaïche, 2018). Loss of epithelial cell adhesion, downregulation of E-cadherin and integrins, and upregulation of N-cadherin and vimentin (markers of the mesenchymal phenotype) are key to the regulation of EMT (Gheldof and Berx, 2013; Zhitnyak et al., 2020). For example, a shift from E-cadherin to N-cadherin expression and elevated EMT levels were observed in lung tissue samples with bronchopulmonary dysplasia and in isolated AT2 cells (Yang et al., 2014). Moreover, AJ transformation occurs as a key step in EMT, allowing epithelial cells to stratify or separate and acquire a mesenchymal phenotype (Amack, 2021). At the onset of EMT, the actin-binding protein EPLIN is phosphorylated, leading to decreased stability of the peripheral actin bundle and its increased tendency for depolymerization (Zhitnyak et al., 2020). E-cadherin colocalizes with peripheral actin bundles in linear AJs of epithelial cells, and the stable linear AJs undergo dramatic changes with the relaxation of cell–cell boundaries upon depolymerization of actin filaments. These changes include the appearance of prominent punctate AJs and pseudopods near punctate AJs, which are regulated by Arp2/3-mediated assembly of branching actin networks. Here, actin reaccumulates and behaves as straight actin fibers, eventually generating centripetal forces at the cell–cell contact site to facilitate cell separation (Zhitnyak et al., 2020) (Figure 2). Therefore, the transformation of AJs during EMT and the weakening of cell–cell adhesion are dependent on the assembly and disassembly of actin filaments.

The proteins controlling the actin cytoskeleton are also altered during EMT to coordinate the motility of mesenchymal cells (Spiering and Hodgson, 2011). The inactivation of LIM domain kinase 2 interferes with the polymerization of actin stress fibers accompanied by reduced phosphorylation of cofilin during endodermal differentiation (He et al., 2021). The deletion of STRIP1 interferes with mesodermal cell actin assembly and disassembly, leading to impaired formation of cell protrusions and reduced rate of focal adhesion formation in mesenchymal stromal cells, thereby affecting their migration ability during EMT and ultimately causing severe defects in embryonic development (Bazzi et al., 2017). In addition, Cdh6 is also an important determinant of cellular actin force production in EMT; it promotes the detachment of the apical tail of NCCs by facilitating the polymerization of apical tail-stabilized actin (Sun et al., 2021). Actin dynamics during EMT are also regulated by several signaling molecules, including Rac1 and Cdc42, which control actin polymerization and protrusion formation at the cell front and Rho-regulated contraction at the posterior edge of the cell (Amack, 2021).

Overall, actin depolymerization relaxes and softens epithelial cortical actin, changes cell shape, and shortens intercellular adhesion junctions during the initial phase of EMT. This period is accompanied by a decrease in E-cadherin and an increase in N-cadherin concentrations (Clay and Halloran, 2014). However, the molecular mechanism of the upregulated N-cadherin remains unclear. In addition, actin polymerization reorganizes the cytoskeleton, further enabling the cells to acquire a migratory phenotype (Hosseini et al., 2020). Notably, the EMT process can alter actin turnover (polymerization and depolymerization) by directly inducing the activity of the cytoskeleton regulatory proteins Rac1 and RhoA (Bellovin et al., 2005; Huang et al., 2014; Hosseini et al., 2020). However, the downstream pathways of Rac1 and Rho that regulate cortical actin assembly remain elusive.

## 5 Role of actin monomers and polymers in tissue and organ development

### 5.1 G-actin isoforms in the development

Various G-actin types aggregate in different proportions to form F-actin with various characteristics to participate in the development of tissues and organs (Kiuchi et al., 2011). Actin is highly conserved, and any mutation during development may have a significant impact on its structure and function (Labasse et al., 2022).

ACTA1 is the major isoform in the skeletal muscle, and mutations in the terminal stop codon of ACTA1 may lead to the elongation of skeletal α-actin, resulting in defects in muscle tissue development (Lornage et al., 2020). ACTA1 mutations affect the hinge region of the central portion of skeletal α-actin and promote the abnormal accumulation of actin bundles in the nucleus (Labasse et al., 2022). Recently, Winter JM et al. showed that ACTA1 mutation leads to the phosphorylation of the highly conserved Gly50 residue 2 in the α-actin substructure. Consequently, the rest of the actin binds to this structural domain, leading to aberrant actin nucleation in the skeletal muscle cells (de Winter and Ottenheijm, 2017). In addition, ACTA1 mutations cause abnormal localization of LINC protein, nesprin-1, nesprin-2, and laminin A/C in muscle cells, which directly or indirectly disrupt the F-actin crosstalk between the nucleus and cytoskeleton and increase the perinuclear gap, leading to defects in nuclear polarization and motility (Pfisterer et al., 2017). In addition, mutations in ACTA1 are associated with three congenital myopathies, namely, congenital fibrous disproportion, nemaline myopathy, and central core disease (Agrawal et al., 2004; Goebel et al., 2006; Nowak et al., 2013). For example, nemaline myopathy manifests as dysfunctional muscle ganglion contraction (Winter et al., 2016; Joureau et al., 2018). Moreover, Acta1-knockout mice show muscle weakness and even death within 9 days after birth; however, elevated expression of Acta2 and Actc1-encoded actin were observed in the skeletal muscle of these mice, which could not fully compensate for the developmental defects caused by the loss of skeletal α -actin (Crawford et al., 2002).

ACTA2, another isoform of α-actin, plays an important role in the contractility of myofibroblasts, mainly in the microfilament bundles of smooth muscle cells. Alterations in ACTA2 may lead to thoracic aortic disease (Milewicz et al., 2017). ACTA2 expression was upregulated in the abnormal morphogenesis of human fetal lung tissue proximal to the epithelium, along with a significant decrease in the proportion of SOX9-positive cells. This led to morphological hypertrophy of smooth muscle cells (SMC) (Danopoulos et al., 2018), thereby suggesting that ACTA2 and its transcription factors, such as SOX2 and SOX9, play important roles in the formation of human fetal epithelial branches by regulating SMCs. ACTA2-knockout mice were viable and morphologically normal but showed defects in vasoconstriction and blood pressure regulation (Schildmeyer et al., 2000), suggesting that ACTA2-encoded α-smooth muscle actin has a relatively mild defective phenotype.

ACTB encodes β-actin, which plays a role in cell adhesion, contraction, and migration. Vera et al. found that β-actin was predominantly present in the stress fibers close to the substrate in fibroblasts. β-actin was preferentially distributed at the cell bases and in lateral cell contact areas in epithelial cells. Co-localization of β-actin with VASP was observed during fibroblast migration and spreading in plate pseudopods and focal adhesions. Silencing of β-actin resulted in a marked decrease in actin stress fibers and an increase in mean cell area in fibroblasts, whereas its overexpression increased protrusions and cell migration area (Dugina et al., 2009). β-Actin accumulation was observed in actively growing structures, such as growth cones and axon bundles, in neuronal development. In the adult cerebellar cortex, β-actin was preferentially present in dendritic spines (Micheva et al., 1998). Drastic reduction of β-actin altered cell shape, migration, and proliferation, thereby impairing brain and heart development (Cuvertino et al., 2017). In addition, the knockdown of the ACTB gene in developing mouse embryos resulted in embryonic death, further suggesting that β-actin is essential for early embryonic development (Bunnell and Ervasti, 2010; Bunnell et al., 2011). Davina et al. hypothesized that the deficiency of β-actin affected the function of cadherin-11 and N-cadherin, preventing NCCs from completing normal EMT and enhancing the apoptotic rate of NCCs, which ultimately impaired the normal development of embryos (Tondeleir et al., 2014). Interestingly, the nucleotide sequence of ACTB was genetically edited to have the full sequence of ACTB but to encode only the ACTG1 protein in a mouse model because the N-terminal amino acid sequences of ACTG1 and ACTB are very similar. The individual development of this mutant mouse and the migration ability of mouse fibroblasts were normal (Vedula et al., 2017). This may indicate that the essential function of β-actin is more dependent on the nucleotide sequence encoding the protein than on the amino acid sequence.

ACTC1, the major α-actin in the heart, encodes cardiac α-actin and copolymerizes with tropomyosin and troponin to form thin cardiomyocyte contractile filaments that link myocyte Z-disks to myosin. Mice with Actc1 deficiency show embryonic or perinatal lethality and myogenic fiber disorders (Kumar et al., 1997). However, overexpression of Actg2 can rescue perinatal lethality caused by α-cardiac actin deficiency and allow mice to survive into adulthood, but mouse hearts still show hypertrophy and reduced contractility (Vedula and Kashina, 2018). The G247D mutation in ACTC1 led to a loss of its functional phenotype with disrupted actin polymerization, increased myocyte apoptosis, and myofibrillar disintegration observed in human and neonatal rat ventricular cardiomyocytes. This was accompanied by an approximately 20% reduction in cardiomyocyte contractility. Moreover, this mutation in ACTC1 resulted in severe advanced heart failure (Frank et al., 2019). Overall, cardiac α-actin is involved in the polymerization of cardiomyocyte actin filaments and is essential for the maintenance of cardiac contractility and left ventricular dimensions.

ACTG1 encodes cytoplasmic γ-actin, whereas ACTG2 encodes smooth muscle γ-actin. ACTG1 was highly expressed in mammalian cochlear cells (Furness et al., 2005). When the ACTG1 gene was mutated in the NIH/3T3 fibroblast cell line, γ-actin on the cell membrane polymerized itself into a multimer, which prevented the monomer from participating in the assembly of actin stress fibers (Morín et al., 2009). Hiroki et al. further hypothesized that the mutant γ-actin (ACTG1) was unable to polymerize into F-actin, resulting in defective actin cytoskeleton repair and progressive disintegration over time in the hair cells of the cochlea, leading to hereditary hearing loss (Miyajima et al., 2020). Not surprisingly, the mice with knockdown of γ-actin survived into adulthood, although they exhibited a higher incidence of hearing loss (Belyantseva et al., 2009). ACTG2 is specifically expressed in smooth muscle cells of the intestinal and urogenital tracts and plays a role in smooth muscle contraction in these organs (Halim et al., 2016). *In vitro* studies have confirmed that ACTG2 mutants had impaired involvement of γ-actin in aggregation, leading to reduced contractility of their SMCs, which, in turn, caused Hirschsprung disease (Moore et al., 2019). Missense changes in ACTB and ACTG1 were found in Baraitser–Winter syndrome, characterized by malformed brain development (Rivière et al., 2012). Overall, the nature of actin filaments may be altered because of mutations in the actin isoform genes leading to developmental defects.

Previously, some authors have suggested that the knockdown of a single actin isoform did not reduce overall actin levels; this was often compensated by the upregulation of other actin isoforms (Belyantseva et al., 2009) (69). However, in most cases, this compensation could not restore overall function (Vedula and Kashina, 2018), which suggested that each actin isoform has a unique function to meet the developmental needs of the body. Defects in any of the isoforms may lead to changes in the morphology and function of the aggregated F-actin, interfering with aspects, such as motor deformation of the associated cells, and ultimately producing developmental defects in processes, such as organ morphogenesis and tissue remodeling. As such, the understanding of the specific function of each monomer is limited to mouse knockout models and observations of cellular phenotypes, and the specific mechanisms and pathways have not been fully elucidated. Recently, Vedula P et al. found that the actin isoforms upregulated in different actin isoform knockout models were usually the ones with ribosome densities closest to that of knocked-out ones, such as ACTA1 and ACTA2 (Vedula et al., 2017). This finding suggests that only heterodimers with similar ribosome densities may compensate for each other. However, the overall functional diversity that drives actin isoforms is achieved at multiple levels, and elucidating the underlying mechanisms of nucleotide-based actin coding will be one of the major focuses of future research.

### 5.2 Actin polymerization mediates tissue and organ morphogenesis

Actin polymerization and depolymerization are essential for tissue and organ formation during embryonic development (Izadi et al., 2021; Omelchenko, 2022). The large amount of F-actin accumulated in the tails of NCCs, which acquired a migratory phenotype through EMT, whereas actin aggregates did not accumulate in NCCs that did not undergo EMT (Clay and Halloran, 2014). Consequently, defective actin polymerization in NCCs repressed cell migration and affected craniofacial bone formation, pharyngeal arch artery remodeling, and cardiac outflow tract separation in mammals (Liu et al., 2013). However, the exact mechanisms and implications remain unclear. Trim59 deficiency interfered with actin polymerization during the differentiation of embryonic stem cells at the blastocyst stage, resulting in impaired development of mouse proto-intestinal embryos (Su et al., 2018). Cdc42 mediated the induction of actin assembly in mammalian embryonic stem cells by PIP2, and its deficiency caused early embryonic death in mice (Chen et al., 2000). In addition, the netrin/DCC, SLIT/Robo, and ephrin signaling pathways promoted F-actin assembly and polarization through the WAVE/SCAR complex to regulate normal embryo and organ development (Bernadskaya et al., 2012). However, excessive aggregation of F-actin also detrimental to embryonic development. For example, excessive aggregation of F-actin resulted in a significant increase in the nuclear expression of YAP in Hela cells, leading to excessive cell proliferation and organ overgrowth (Sansores-Garcia et al., 2011).

Actin polymerization participated in the formation of swollen protoplasts by nuclei of the sperm and egg in mammalian embryonic development, which were essential for producing fully fertilized eggs (Okuno et al., 2020). Interfering with the assembly of F-actin in protoplasts reduced the expression of genome-activating genes, which resulted in abnormal embryonic development (Shi et al., 2022). Therefore, the stable polymerization of actin is important for early embryonic development. Disruption of actin polymerization by inhibiting serine/threonine protein kinase (LIMK1/2) impaired early embryo division at the fertilized egg stage, and disruption of cortical actin assembly in oocytes at the early mouse embryonic developmental stage reduced the amount of F-actin, failing embryo compaction and blastocyst formation (Gumus et al., 2010; Duan et al., 2018). SMTNL2 of the smooth muscle protein family inhibited coronin-1B, prevented the rapid polymerization/depolymerization of actin, and promoted epithelial morphogenesis by stabilizing actin filaments in 3D-MDCK cells (Hachimi et al., 2021). However, the apical-basal polarization of cells within the embryo was crucial for the differentiation and shaping of different organ tissues at a later stage, which depended on the apical enrichment of F-actin in embryonic cells (Zhu et al., 2021).

F-actin was locally assembled in the growth cone in mammalian neural development, whereas impaired local assembly of F-actin affected the growth of neural synapses (Chia et al., 2016). Moreover, the kinetics of actin assembly was controlled by local Ca^2+^/calmodulin (CaM) and depended on the interaction among different F-actin assembly regulators to secure local actin polymerization and promote the formation of complex neuronal morphology in mammals (Izadi et al., 2021). Additionally, profilin1 promoted apical radial glial cell division by regulating actin assembly and participated in mouse neocortex development (Kullmann et al., 2020). Plexin-B2 maintained the cytoarchitectural integrity of the neuroepithelium by modulating actin assembly, cell stiffness, and intercellular and cell-matrix adhesion during the multicellular development of human embryonic stem cells and neural progenitor cells (Junqueira Alves et al., 2021).

The precise assembly of actin filaments is critical for muscle development. Kank1 regulated myogenic cell differentiation by regulating actin remodeling and cell proliferation in C2C12 progenitor cells (Nguyen and Lee, 2022). Additionally, defects in F-actin assembly contributed to the development of skeletal myopathies and cardiomyopathies. For example, CAP2 played a key role in the maturation of cardiomyocytes by regulating the ratio of α-actin composition in F-actin thin filaments (Colpan et al., 2021), and its abnormal expression induced depolymerization of actin filaments and defects in striated muscle development (Iwanski et al., 2021).

Actin assembly played an important role in the development of several other tissue or organ structures. α-Parvin controlled epidermal morphogenesis and hair follicle development by promoting integrin-mediated adhesion and actin assembly in keratin-forming cells (Altstätter et al., 2020). The involvement of actin rearrangement in the development of epithelial buds was observed in the organ culture of the mouse embryonic submandibular gland (Kim et al., 2021). Tln1 deficiency induced the disruption of F-actin rearrangement, resulting in the disturbance of vascular endothelial cell elongation and disruption of cell–cell junctions in vascular morphogenesis (Chau et al., 2022). Additionally, thick and dense actin stress fibers were observed in palatal synaptic mesenchymal cells during mouse embryonic palate development (Chiquet et al., 2016). Interestingly, we conditionally knocked out β-catenin in the mouse palatal mesenchyme and observed delayed palatal elevation with cleft palate in mice, which was caused by the reduced polymerization of actin in the palatal mesenchyme of mutant embryos (Pang et al., 2021). This indicated that β-catenin in the palatal mesenchyme may regulate palatal elevation by mediating actin polymerization. Similarly, Wang et al. also observed a decrease or increase in α-actinin-4 counterpart after mesenchymal-specific inactivation or overexpression of β-catenin and proposed that α-actinin-4 might be a target of β-catenin in regulating F-actin (Wang X. et al., 2022). Recently, Liu W et al. conducted transcriptome analysis and suggested that Saa3 and Cxcl5 may be the intermediate targets for β-catenin in the regulation of F-actin rearrangement in mouse embryonic palatal mesenchymal cells. However, no differential expression of these genes was observed in mice *in vivo* (Liu et al., 2022). Taken together, these findings clarify the crucial role of β-catenin-mediated actin polymerization and rearrangement in palatal development; however, the specific mechanisms need to be further explored.

Actin polymerization also plays a prominent role in the development of non-mammals. For example, Rho controlled force asymmetry to drive morphogenesis by promoting the formation of actin stress fibers in *Drosophila* embryonic epithelium (Ong et al., 2019), and Arf-GEF Steppke promoted actin polymerization during *Drosophila* dorsal closure and completed tissue sealing (Romero et al., 2020). Twinfilin1 deficiency prevented the polymerization and depolymerization of cytoplasmic actin, resulting in a defective proto-intestinal embryo formation in the African clawed frog (*Xenopus*) (Devitt et al., 2021).

Collectively, these findings suggested that the balance of actin polymerization and depolymerization is fundamental for tissue and organ morphogenesis in vertebrates, including mammals. Failure of timely polymerization or depolymerization of actin results in failure to form an intact organism. However, studies on actin polymerization are still limited to animal or cellular phenotypes, and few studies are available on the existence of functional similarities and differences in the F-actin polymerized by different actin monomers, and the unique role of the monomer itself. Table 1 lists the role of actin isoforms in development and disease.

**TABLE 1:** Role of six actin isoforms in development and disease.

Actin isoform	Expression location	Associated diseases	Mechanism	Abnormalities in mouse knockout models	References
ACTA1	Skeletal and heart muscles	1. Congenital fibrous disproportion	Disruption of actin polymerization, leading to defective cell nuclear polarization and cell migration	Mice showed muscle weakness and finally died	Crawford et al. (2002), Agrawal et al. (2004), Goebel et al. (2006), Nowak et al. (2013), Winter et al. (2016), de Winter and Ottenheijm (2017), Pfisterer et al. (2017), Joureau et al. (2018), Lornage et al. (2020)
		2. Nematode myopathy			
		3. Central core disease			
ACTA2	Smooth muscle	Thoracic aortic disease	Disruption of the morphology of the myofibroblasts and their ability to contract	Embryos survived with defective vasoconstriction	Schildmeyer et al. (2000), Milewicz et al. (2017), Danopoulos et al. (2018)
	Fetal lung tissue				
ACTB	Almost all cells	Defects in brain, heart, and early embryonic development	Impairment of the ability of cells to deform, migrate, and proliferate	Embryonic lethality in mice	Micheva et al. (1998), Dugina et al. (2009), Bunnell and Ervasti (2010), Bunnell et al. (2011), Tondeleir et al. (2014), Cuvertino et al. (2017), Vedula et al. (2017), Moore et al. (2019)
		Baraitser–Winter syndrome	Disrupts EMT of neural crest cells		
ACTC1	Cardiac muscle cells	Advanced heart failure	Disruption of actin polymerization and increased apoptosis in cardiac myocytes	Embryonic or perinatal lethality and myogenic fiber defects, ACTG2 overexpression rescued perinatal lethality	Kumar et al. (1997), Vedula and Kashina (2018), Frank et al. (2019)
		Familial atrial septal defects			
ACTG1	Cochlear cells	Hereditary hearing loss	Failure of γ-actin to polymerize into F-actin, resulting in defective actin cytoskeleton repair and progressive breakdown	/	Furness et al. (2005), Morín et al. (2009), Moore et al. (2019), Miyajima et al. (2020)
ACTG2	Smooth muscle cells of the intestinal and urogenital tracts	Hirschsprung disease	Disruption of actin polymerization and reduction of smooth muscle cell contractility	Survived into adulthood but most had hearing defects or even hearing loss	Belyantseva et al. (2009), Halim et al. (2016)
		Baraitser– Winter syndrome			

### 5.3 Nuclear actin in the early stages of development

F-actin filaments in tissue cells are ordered and polarized at multiple cell boundaries to coordinate overall intercellular forces during tissue and organ development (Sánchez-Corrales and Röper, 2018). Interestingly, the functional actin is present in the nucleus (Plessner and Grosse, 2019). Some authors have identified actin in the nucleus of African clawed frog oocytes. Nuclear F-actin has only been observed in certain specific cases, such as in mouse embryonic fibroblasts. High concentrations of G protein-coupled receptors and calcium in these fibroblasts promoted the assembly of nuclear F-actin in chromatin organization to ensure timely cellular responses to the external environment. In addition, the upregulation of actin polymerization in megakaryocytes promoted the release of acute leukemia protein to enhance the transcriptional activity of serum response factor (Baarlink et al., 2013; Wang et al., 2019). Nuclear F-actin is responsible for maintaining the nuclear structure of African clawed frog oocytes and early embryos (Bohnsack et al., 2006). Oocyte nuclei >10 µm in diameter required stable nuclear F-actin as a scaffold against gravity (Feric and Brangwynne, 2013). In mammals, F-actin accumulated in pronuclei of fertilized eggs of mice embryos, and perturbations in nuclear actin dynamics in fertilized eggs resulted in the dysregulation of genes associated with mouse embryo development (Okuno et al., 2020).

Some authors have suggested that the nucleus contained mainly or only β-actin (Hofmann et al., 2004; Falahzadeh et al., 2015). However, Migocka-Patrzałek M et al. found that the nucleus also contained a small amount of γ-actin (Migocka-Patrzałek et al., 2015). Although the two isoforms differ only in the four encoded amino acids, they have different physiological functions. Moreover, they differ in polymerization and depolymerization kinetics. γ-Actin polymerizes slowly and forms stable polymers. In contrast, β-actin shows active polymerization and fast nucleotide exchange (Bergeron et al., 2010). However, the specific role of nuclear actin isoforms (with different polymerization rates and filament stability) on nuclear transcription and cellular physiological functions have not been extensively investigated. Moreover, it is not known whether nuclear actin only contains β-actin and γ-actin, and this may be the main topic of future work on nuclear actin.

## 5 Conclusion and perspectives

Actin is one of the most abundant intracellular proteins, and its polymerization and depolymerization are essential for various physiological activities in the cell. Several researchers have elaborated on the critical roles of actin monomers and polymers in regulating cell, tissue, and organ morphogenesis in development. However, some questions are still unanswered. For example, how embryonic cells constantly and transiently respond to the external environment changes to ensure the smooth shaping of tissue and organ morphology by regulating actin assembly and disassembly. It is still unknown whether different isoforms of G-actin in the cell have their unique effects on the function of the polymerized actin filaments. Additionally, neither the phenotypic observations nor the mechanisms of actin defects in mice have been adequately studied. Recently developed *in vivo* imaging and labeling techniques have allowed us to observe the dynamic actin cytoskeleton in living mammalian embryos (Yan et al., 2019; Padhan et al., 2022). Future research should focus on cellular and actin biomechanics to understand how actin polymerization extends its effects on cells to organs and embryos (Tran and Kumar, 2021; Chatterjee et al., 2022). Continuous research in this field will unravel the complex functions of actin in shaping a complete organism in the future.
